# Leveraging wastewater surveillance for managing the spread of SARS-CoV-2 and concerned pathogens during FIFA World Cup Qatar 2022

**DOI:** 10.1016/j.heliyon.2024.e30267

**Published:** 2024-04-26

**Authors:** Shimaa S. El-Malah, Jayaprakash Saththasivam, Arun K. K, Khadeeja Abdul Jabbar, Tricia A. Gomez, Sara Wahib, Jenny Lawler, Patrick Tang, Faheem Mirza, Hamad Al-Hail, Khalid Ouararhni, Thasni K. Abdul Azis, Laith Jamal Abu Raddad, Hiam S. Chemaitelly, Hussein A. Abu Halaweh, Sara Khalife, Roberto Bertollini, Khaled A. Mahmoud

**Affiliations:** a Qatar Environment and Energy Research Institute (QEERI), Hamad Bin Khalifa University, Qatar Foundation, P. O. Box 34110, Doha, Qatar; b Department of Pathology, Sidra Medicine, Doha, Qatar; c Genomics Core, Hamad Bin Khalifa University, Qatar Foundation, Doha, Qatar; d Infectious Disease Epidemiology Group, Weill Cornell Medicine-Qatar, Cornell University, Doha, Qatar; e Drainage Network Operation & Maintenance Department, Public Works Authority, Doha, Qatar; f Ministry of Public Health, Doha, Qatar

**Keywords:** COVID-19, WBE, Large gathering surveillance, FIFA WC Qatar 2022, Wastewater, SARS-CoV-2

## Abstract

Wastewater-based epidemiology (WBE) has been proven effective for the monitoring of infectious disease outbreaks during mass gathering events and for timely public health interventions. As part of Qatar's efforts to monitor and combat the spread of infectious diseases during the FIFA World Cup Qatar 2022™ (FWC’22), wastewater surveillance was used to monitor the spread of SARS-CoV-2, human enterovirus, and poliovirus. The screening covered five major wastewater treatment plants servicing the event locations between October 2022 and January 2023. Viruses were concentrated from the wastewater samples by PEG precipitation, followed by qRT-PCR to measure the viral load in the wastewater. As expected, SARS-CoV-2 and enterovirus RNA were detected in all samples, while poliovirus was not detected. The concentration of SARS-CoV-2 was correlated with population density, such as areas surrounding the World Cup venues, and with the number of reported clinical cases. Additionally, we observed temporal fluctuations in viral RNA concentrations, with peak levels coinciding with the group stage matches of the FWC’22. This study has been useful in providing public health authorities with an efficient and cost-effective surveillance system for potential infectious disease outbreaks during mega-events.

## Highlights

• Wastewater surveillance was utilized to monitor SARS-CoV-2, enterovirus, and poliovirus during the FIFA World Cup Qatar 2022.
• SARS-CoV-2 and enterovirus RNA were detected in all samples, while poliovirus was not detected.
• Sequencing revealed that Omicron XBB.1 was the predominant subvariant during the games period.
• Wastewater-based epidemiology offers an efficient, economical way to detect disease outbreaks during major events.

## Graphical abstract

**Figure:**
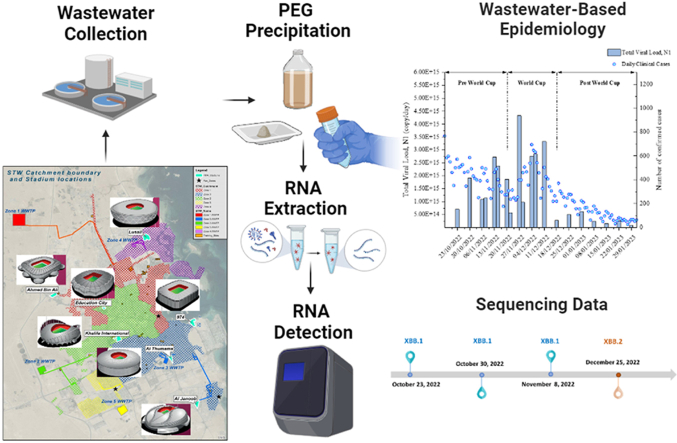

## 1 Introduction

Qatar, with a small area of 11,521 km^2^ and a population of slightly over 2.6 million, made history by hosting the momentous FIFA World Cup Qatar 2022™ (FWC’22) [1,2]. The event which was held between 20 November to December 18, 2022 marked the first instance where all eight stadiums located in close proximity hosted over 1.7 million attendees, including fans, staff, players, and media. This was a major challenge for a small state to accommodate almost double the current population, especially during the COVID-19 pandemic. The WHO usually works with local health authorities and governments to manage disease control during mass gathering events [3]. Sporting events have been widely impacted by COVID-19, leading to many of them taking place without spectators [4]. For example, the International Olympic Committee (IOC) decided to postpone the 2020 Tokyo Summer Olympics and Paralympics [5,6]. The event was finally held from July to September 2021. Despite the lack of rigorous risk assessment, COVID-19 transmission within the Olympic arena and village seemed to be relatively controlled, suggesting the effectiveness of measures such as adopting the bubble system, regular COVID-19 screening, and extensive vaccination [7,8].

During FWC’22, Qatar authorities decided to relax prescreening measures and vaccination requirements for international fans to maximize spectator experience during the tournament. To mitigate the associated risks, it was important to adopt alternative surveillance systems to monitor the possible spread of the infection during the event. The WHO has recommended the use of wastewater-based epidemiology (WBE) as a support measure to monitor the spread of SARS-CoV-2 at a population level. This includes assessing the presence or absence of infection, monitoring developing trends over time, and tracking variants [9]. Also, the WHO recommended keeping the usual preventive measures including disinfection, face masks, and practicing social distancing whenever possible [10]. WBE surveillance offers a more cost-effective and efficient approach for monitoring diseases on a broad scale when compared to clinical or epidemiological surveillance methods [11]. When combined with rapid detection laboratory techniques like genomics and biosensors, utilizing WBE as a preventive measure can offer a vital tool in anticipating potential disease outbreaks [12,13], while targeted surveillance (for example, at airport hubs) can provide further information on disease spread trajectories surrounding global travel for sporting mega-events [14]. In the past, WHO had adopted WBE to screen polio virus and identify high-risk areas under the Global Polio Eradication Initiatives. It is now being utilized to monitor the spread of SARS-CoV-2 and its variants [15]. Moreover, WBE has gained recognition as the primary data source for detecting the circulation of Enteroviruses [16]. This approach also facilitates the detection of other pathogens transmitted through the fecal-oral route, making it possible to consider all infected individuals, both asymptomatic and symptomatic [17].

WBE was used during the Tokyo 2020 Olympic and Paralympic Games and SARS-CoV-2 RNA was detected in 33.8 % of 690 samples collected using passive and grab sampling methods [18]. Importantly, these positive detections occurred even in areas where no positive COVID-19 cases had been reported through clinical testing [19]. The first study that used WBE for monitoring SARS-CoV-2 in Qatar's municipal wastewater started during the pandemic's first wave in 2020 [20]. Another case study was reported in Qatar with the application of a new human RNase P normalization method for a more accurate estimation of the infected population as well as the identification of new variants of concern during 2021 [21].

In this study, we have successfully employed wastewater surveillance to monitor the spread of SARS-CoV-2, human enterovirus, and poliovirus outbreaks during Qatar FWC’22. The screening involved biweekly sample collection from five major wastewater treatment plants covering the Whole genome sequencing was used to identify the circulating variants during the event.

## 2 Materials and methods

### 2.1 Wastewater sample collection and initial processing

**Fig. 1:**
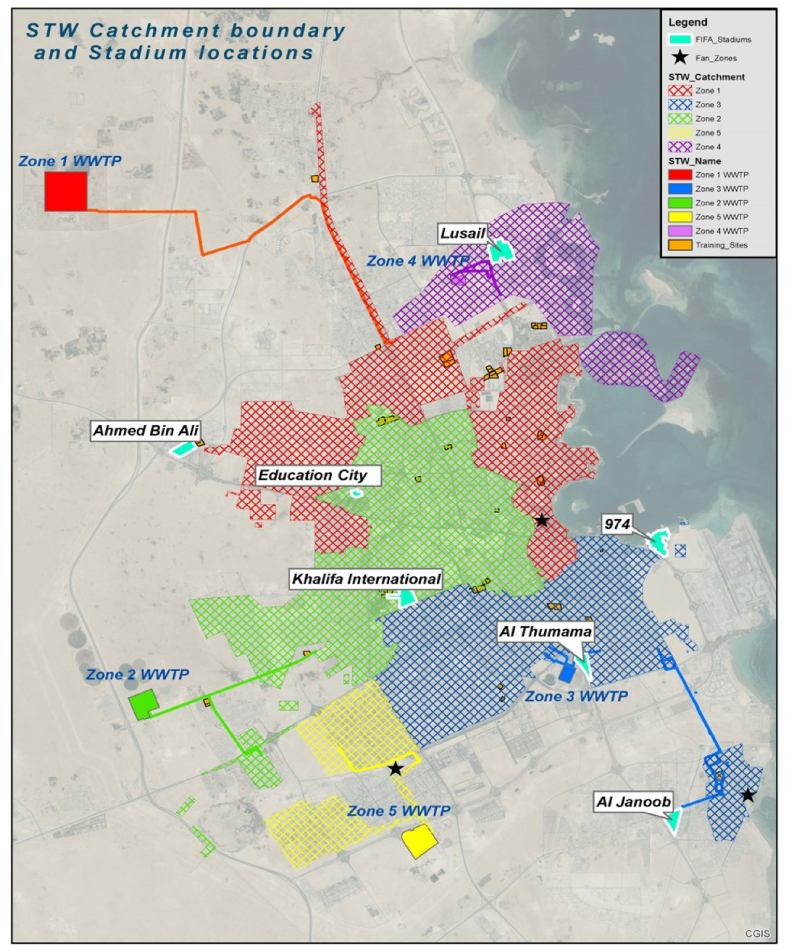
Location of the FWC’22 stadiums, fan zones, and respective wastewater treatment plants in Qatar.

A total of 105 untreated wastewater samples were collected from five major wastewater treatment plants (WWTPs) in Qatar between October 2022 and January 2023, as depicted in Fig. 1. The specific WWTPs included in the study have been designated as Zones 1 to 5. Samples are collected weekly during the pre-event period. The sampling frequency was increased to twice a week from November to the first week of December 2022 to increase the number of data points. This adjustment was particularly crucial as the population in Qatar was expected to peak during the games. Composite samples were collected over 24 h in sterile amber glass bottles and were heat treated at 56–58 °C in a water bath for 30 min to reduce the infection risk for laboratory staff [20,22]. The samples were transported on ice to the laboratory for further processing within 2 h of collection and residual samples were stored at −80 °C as reference.

### 2.2 Viral concentration

Viral fragments were concentrated from the samples using the PEG protocol as described previously [23,24]. Briefly, 25 mL of glycine buffer (0.05 M UltraPure™ Glycine (Invitrogen™, by Thermo Fisher Scientific, Cat. No. 15527013, CA, USA) and 3 % Bovine Serum Albumin (BSA) (Sigma-Aldrich, Cat. No. A9418, Louis, MO, USA) was added to 200 mL of untreated wastewater. The mixture was incubated at 4 °C for 2 h with gentle shaking at 200 rpm. Then, the samples were centrifuged at 4500×*g* for 30 min at 4 °C to remove large debris and bacterial cells. Following centrifugation, the supernatant was transferred into a sterile container. PEG 8000 (100 g/L) (Sigma-Aldrich, CAS. No. 25322-68-3, Louis, MO, USA) and NaCl (22.5 g/L) (Sigma-Aldrich, CAS. No. S7653, Louis, MO, USA) were then added to the supernatant. The solution was gently inverted by hand to dissolve the PEG and NaCl. The mixture was incubated overnight at 4 °C with continuous agitation at 100 rpm. The sample was then centrifuged for 90 min at 13,000×*g* and 4 °C, and the supernatant was removed. The sample was centrifuged again for 10 min at 13,000×*g* with slow braking (brake set at 3 out of 9). The pellet containing the concentrated viruses was eluted by adding 1 mL of DNA/RNA Shield™ (Zymo Research, Irvine CA, USA Cat. No. R1100-250) and stored at −80 °C until further processing. The concentration method was performed in triplicate, along with a negative control consisting of treated wastewater effluent.

### 2.3 Extraction of viral RNA and qRT-PCR analysis

Viral RNA was extracted from 200 μL of the pellet using the QIAamp Viral RNA Mini Kit (Qiagen, Germantown, Maryland, USA Cat. No. 52906) according to the manufacturer's protocol. The viral RNA was eluted in 40 μL of nuclease-free water and stored at −80 °C prior to qRT-PCR. The identification and quantification of SARS-CoV-2 in the extracted viral RNA was assessed using SARS-CoV-2 (2019-nCoV) CDC qPCR Probe Assay Research Use Only (RUO) kit (Integrated DNA Technologies, IDT, Coralville, IA, USA Cat. No. 10006713) and Super Script™ III Platinum™ One-Step qRT-PCR Kit (Thermo Fisher, USA; Cat. No. 11732088) on Quant Studio™ 5 Real-Time PCR System (Applied Biosystems, CA, USA) [18,25]. SARS-CoV-2 (2019-nCoV) CDC RUO Plasmid Controls (Integrated DNA Technologies, IDT, Coralville, IA, USA Cat. No. 10006625) was used as a positive control. All qRT-PCR amplifications were performed in 20 μL reaction mixtures each containing: 3.6 μL nuclease-free water, 1 μL of each combined primers and probe mix, 0.4 μ L Super Script™ III Platinum One-Step Enzyme, 10 μL Super Script™ III Platinum (2X) Reaction Mix, and 5 μL extracted viral RNA. Cycling conditions: reverse transcription at 55 °C for 15 min, initial denaturation at 95 °C for 2 min, denaturation at 95 °C for 15 s, and extension at 60 °C for 60 s for 45 cycles. Instrument setting: detector (FAM) and Quencher (none), the samples were tested in triplicate. Detection of enterovirus was conducted as previously described [26]. Poliovirus testing was performed as per the WHO/CDC pan-poliovirus protocol [27]. PCR detection of the human RNase P gene was used to control sample adequacy. For the detection of enterovirus and poliovirus, samples were tested in duplicate by qRT-PCR on the ABI 7500 Fast Real-Time PCR System (Thermo Fisher Scientific, Waltham MA, USA) using Taq Path 1-Step qRT-PCR master mix (Thermo Fisher Scientific).

### 2.4 Sequencing of virus RNA fragments

Viral RNA samples with SARS-CoV-2 qRT-PCR Ct values below 30 were processed for Whole genome sequencing (WGS). First, samples were subjected to a second purification using 1.2x RNAClean XP beads (Cat#A66514, Beckman Coulter). Two cDNAs were synthesized from each sample and amplified simultaneously, producing 400 bp amplicons, using two different primer pools from the Artic V5.3.2 NCOV-2019 Panel (Cat#10016495). Two amplicon products from each sample were pooled and purified using Beckman 0.8x AMPure XP beads (Cat # A63882, Beckman Coulter). The quality of the amplicons generated was checked on an Agilent 2100 Bioanalyzer system and quantified using the Qubit system.

The purified amplicons were used for Illumina DNA prep library preparation (Cat # 20060059, ILLUMINA) for whole genome sequencing. Briefly, the purified samples were subjected to tagmentation process where Bead Linked Transposomes (BLT) were used to fragment and tag DNA with adapter sequences. This follows a series of washes and elution of tagmented and purified DNA for library amplification where unique indexes were incorporated to each sample. The quality of the generated libraries was checked on an Agilent 2100 Bioanalyzer system and quantified using the Qubit system. Libraries that passed quality control were pooled and sequenced on the NEXTSEQ2000 system (Illumina platform) with a minimum of 5 million paired end reads (2x100 bp) per sample.

### 2.5 Viral load estimation

When monitoring the outbreak of viruses using wastewater surveillance, it is important to assess the spread of the virus by tracking viral load (copy/day) rather than solely relying on viral RNA concentration (copy/L) directly obtained from qRT-PCR analyses. Calculating viral load (equation (1)) by considering the flow rate allows for comparisons between different sewage treatment plants, especially if they are operating at different capacities. Additionally, viral load provides a more accurate correlation with the possible number of infected people served by the wastewater treatment plant.

(1) $$\text{Viral}\;\text{Load}=C_{\text{RNA}\left(\text{WWTP}\right)}\times F$$

where $C_{\text{RNA}\left(\text{WWTP}\right)}$ represents the measured RNA concentration (copy/L) of the SARS-CoV-2 or human enterovirus at the inlet of WWTP, while F denotes the volumetric flow rate of the WWTP (L/day) respectively. The total viral loads were calculated by summing the viral load of the five major WWTPs.

The total population (P) in the country can be estimated by summing the ammonia loading from these five major zones:

(2) $$P=\sum_{i}^{5}\left(\frac{C_{\text{NH}4\left({\text{WWTP}}_{i}\right)}\times F_{i}}{M_{\text{NH}4-N}}\right)$$

Here, $C_{\text{NH}4}$ = Ammonium concentration in the WWTP inlet (mg/L)

$M_{\text{NH}4-N}$ = Daily average of ammonium excreted per person (=6000 mg/day/person) [28,29]

## 3 Results and discussion

**Fig. 2:**
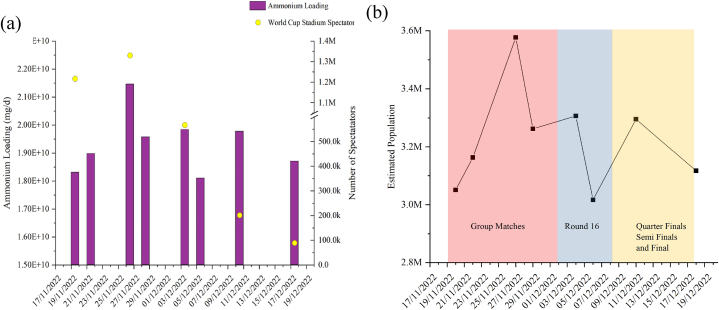
(a) Comparison of ammonia loading trend against the aggregate weekly attendance and (b) estimation of total population during the entire event period.

The concentrations of SARS-CoV-2 RNA, polio, and enterovirus were measured from mid-October 2022 until the end of January 2023, during which a total of 105 composite wastewater samples were collected over a 24 h period from five WWTPs in Qatar (see Fig. 1). The total population during different phases of the event was estimated by measuring the amount of ammonia being produced. This estimate was then compared to the aggregate weekly attendance figures announced during matches. As shown in Fig. 2 (a), both total amount of produced ammonia across the five zones and attendance at the games showed a comparable trend. As depicted in Fig. 2b, the estimated total population in Qatar approached 3.6 million during the group matches, based on ammonia loading. A decrease in the population can be observed thereafter, with the population closing in on 3.1 million towards the end of the World Cup [30].

**Fig. 3:**
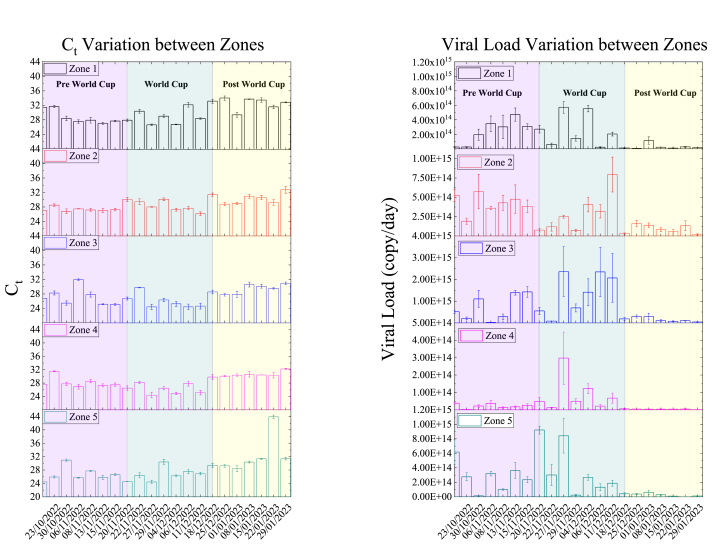
(a) The SARS-CoV-2 Ct values and (b) the viral load of the raw wastewater of Qatar's five zones representing major WWTPs. The error bars in the bar graphs represent standard deviations, which were calculated from duplicate measurements.

The results of qRT-PCR analysis exhibited concordance between the N1 and N2 assays. Fig. 3a illustrates the detection of SARS-CoV-2 RNA in every wastewater sample, indicating positive qRT-PCR signals within the detectable range with Ct values below 35 cycles. Moreover, all WWTPs recorded their highest viral loads during the World Cup, with a significant drop observed towards the end of the event (Fig. 3b). The highest viral load was noted at Zone 3 (2.35x10^15^ copies/day), which served three World Cup stadiums and one fan zone. Additionally, Zone 3 is one of the largest facilities, operating at an average capacity of 200,000 m^3^/day. Conversely, Zone 4 consistently exhibited the lowest viral load throughout the sampling period, which is unsurprising given its status as the smallest among the five zones studied here, operating at a mere capacity of approximately 15,000 m^3^/day. Nevertheless, a notable relative increase was observed during the games, most likely because Zone 4 houses Qatar's largest stadium, boasting a seating capacity exceeding 80,000. Additionally, Zone 4 was a highly popular tourist attraction during the tournament. A similar pattern emerges in Zone 5, where viral loads peaked during the group stage matches. Interestingly, despite not servicing any World Cup stadiums directly, this increase in viral load can be attributed to its association with one fan zone and the majority of hospitality teams housing.

**Fig. 4:**
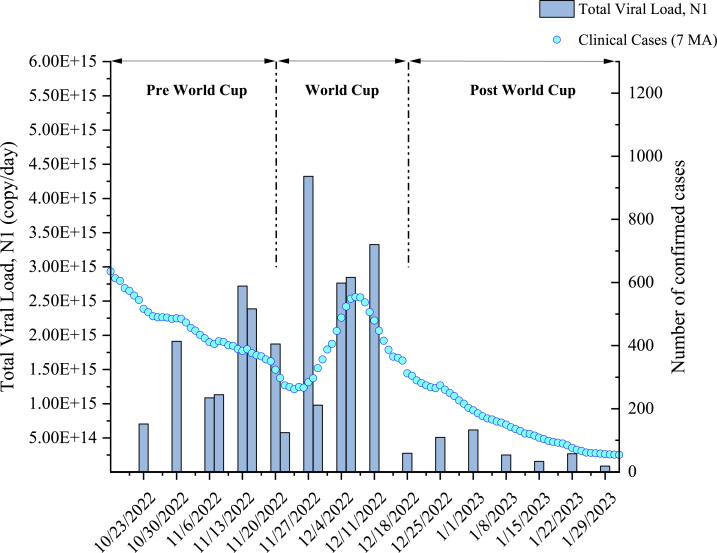
Comparison of total viral load of SARS-Cov-2 against 7 days moving average of daily positive clinical cases during the FWC’22 period.

The temporal variation in the total SARS-Cov-2 viral load in the five major zones is shown in Fig. 4. The figure offers a more comprehensive insight into the potential spread of Covid-19 throughout the entire country, given that these zones represent most of the population (over 95 %). A distinct upward trend in viral load became evident during the pre-World Cup period. Notably, even before the games commenced, wastewater viral load was on the rise, despite a decline in reported clinical cases of COVID-19. The decline in clinical cases is attributed to the major changes in testing policy that went into effect in Qatar starting from November 1, 2022, right before the World Cup [[31], [32], [33]]. The testing rate decreased from about 5 % of the population being tested per week to less than 1 % of the population being tested per week. A notable surge in COVID-19 clinical cases in Qatar during the third and fourth weeks of FWC’22, aligning with the schedule of group-stage matches, as opposed to the earlier weeks. Subsequently, the reported clinical cases dropped significantly, mirroring the decline of SARS-CoV-2 RNA in the wastewater [34]. Cross-correlation analysis (Table S3 and Fig. S1) identified a significant positive correlation (r = 0.53234) at lag 0, indicative of a moderate in-phase relationship between clinical data and viral load. The results suggest that the viral load reading from the wastewater analysis appeared to simultaneously fluctuate with the trend of the clinically reported cases during the games. This suggests that wastewater-based epidemiology (WBE) could be a valuable alternative for outbreak disease monitoring. During the post-World Cup period, a sharp and sustained decline in viral load levels is noticeable. This decline can primarily be attributed to shifts in population dynamics. As the World Cup concluded and the year-end vacation season began, a substantial portion of the local population left the area. Additionally, with the decrease in tourism-related activities after the event, there were fewer potential sources of the virus in the wastewater, including both local and visiting populations. These combined factors played a pivotal role in the observed decline in viral load.

**Fig. 5:**
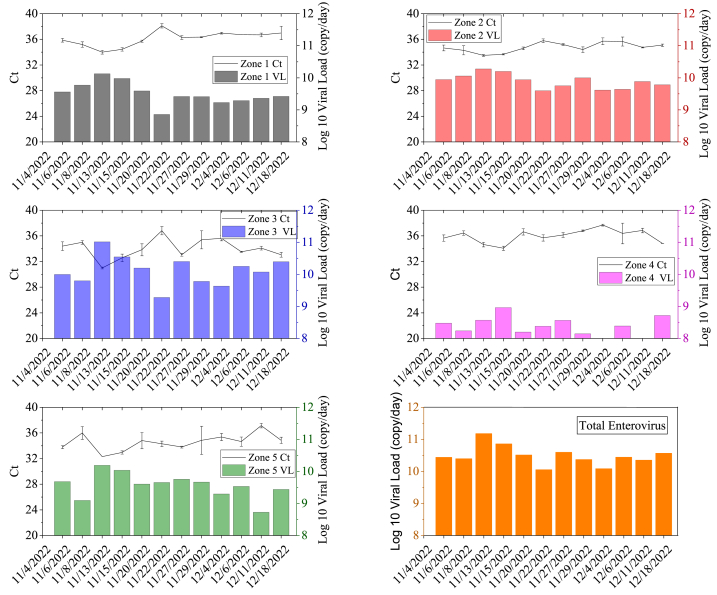
The Ct value and viral load of enterovirus RNA in wastewater samples.

Human enterovirus RNA was detected in all wastewater samples while poliovirus was not detected. The limit of detection for poliovirus was determined to be 225 genome copies per 1 mL of pasteurized wastewater. The enterovirus viral load for each zone and entire Qatar is shown in Fig. 5. Similar to SARS-CoV-2, there was a higher concentration of enterovirus in Zone 3 where there was the greatest number of visitors and fans. There was also a sharp increase in wastewater enterovirus concentration in all zones, but especially Zone 3, coinciding with the large influx of visitors at the beginning of the event. .

**Fig. 6:**
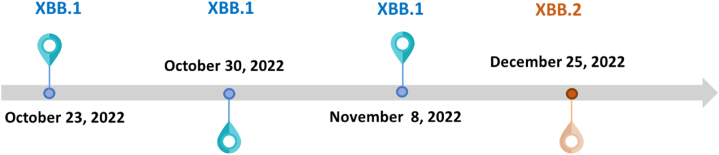
Subvariant of Omicron lineage detected during the during the FIFA World Cup 2022 period.

To improve the detection of SARS-CoV-2 variants in wastewater samples, weekly samples from the five zones were pooled for sequencing. This increased the amount of viral genetic material (genome coverage), enhancing the success rate of identifying variants in subsequent sequencing steps. The percentage of positive samples displaying specific lineages was determined to be 40 %. Notably, two distinct lineages were successfully identified through the sequencing process. At the end of October (23 and 30) and November 8, 2022, the XBB.1 subvariant within the Omicron lineage emerged as the predominant strain, accounting for 100 % prevalence during that period. Meanwhile, towards the end of December 2022, the subvariant XBB.2 exhibited predominance, assuming prominence during that temporal interval (see Fig. 6). Omicron and its subvariants ranked as the dominant SARS-CoV-2 variant by the end of 2022, circulating globally, with Omicron subvariants XBB.1. and XBB.2 causing almost all COVID-19 infections [35]. Surveillance of SARS-CoV-2 variants in Qatar carried out using viral genome sequencing and multiplex qRT-PCR testing between September 10, 2022, and April 21, 2023, identified XBB and BA.2.75 as the primary Omicron subvariants through testing of random clinical cases [36]. As a result, our findings were consistent with the outcomes of clinical surveillance conducted in Qatar between September 2022 and April 2023 within the timeframe of the FIFA event. This agreement strengthens the reliability of wastewater-based surveillance, confirming that the Omicron subvariants XBB were indeed prevalent and important during that period. However, BA.2.75 was not observed in the wastewater, despite its detection in clinical samples. In situations, where SARS-CoV-2 incidence is low, virus concentrations in wastewater may not be detectable, making it difficult to amplify and sequence its genome. Currently, there are several limitations to the sequencing of SARS-Cov-2 in wastewater. Developing enhanced methodological approaches is necessary to prevent inhibition from substances in wastewater matrices.

## 4 Conclusion

Wastewater-based surveillance has played a pivotal role during the FIFA World Cup 2022 in detecting infections and tracking transmission within a population, regardless of clinical symptoms or socioeconomic biases associated with case reporting. The integration of wastewater surveillance for infectious diseases during mass gatherings has demonstrated its effectiveness in providing timely warnings to host countries and global public health authorities. Additionally, this approach can provide insights to other countries regarding potential pathogens introduced by returning travelers. WBE aligns with clinical surveillance efforts and contributes valuable insights into the prevalence and dynamics of Omicron subvariants.

## Data availability

The data used in this study are available from the corresponding author upon request.

## CRediT authorship contribution statement

**Shimaa S. El-Malah:** Writing – original draft, Methodology, Investigation, Formal analysis, Data curation. **Jayaprakash Saththasivam:** Writing – review & editing, Writing – original draft, Methodology, Investigation, Formal analysis, Data curation. **Arun K. K:** Visualization, Resources, Methodology. **Khadeeja Abdul Jabbar:** Resources, Methodology, Formal analysis. **Tricia A. Gomez:** Visualization, Resources. **Sara M. Wahib:** Visualization, Validation, Resources. **Jenny Lawler:** Writing – review & editing, Supervision. **Patrick Tang:** Writing – review & editing, Validation, Supervision, Investigation. **Faheem Mirza:** Resources, Methodology. **Hamad Al-Hail:** Resources, Methodology, Formal analysis. **Khalid Ouararhni:** Writing – review & editing, Supervision, Investigation. **Thasni K. Abdul Azis:** Resources, Formal analysis. **Laith Jamal Abu Raddad:** Writing – review & editing, Validation, Supervision. **Hiam S. Chemaitelly:** Resources, Formal analysis, Data curation. **Hussein A. Abu Halaweh:** Validation, Resources. **Sara Khalife:** Writing – review & editing, Resources. **Roberto Bertollini:** Supervision, Resources, Project administration. **Khaled A. Mahmoud:** Writing – review & editing, Validation, Supervision, Project administration, Investigation, Funding acquisition, Conceptualization.

## Declaration of competing interest

The authors declare that they have no known competing financial interests or personal relationships that could have appeared to influence the work reported in this paper.

## References

[1] Portal Q.E.-g. (2023). "*About Qatar" on the Qatar E-government portal*. https://hukoomi.gov.qa/en/about-qatar.

[2] Choudhary O.P., Saied A.A., Shafaati M. (2022). FIFA World Cup 2022: bouncing off the infectious balls. Trav. Med. Infect. Dis..

[3] WHO. *Managing health risks during mass gatherings*. 2021 04042024]; Available from: https://www.who.int/activities/managing-health-risks-during-mass-gatherings.

[4] Dergaa I. (2021). Organising football matches with spectators during the COVID-19 pandemic: what can we learn from the Amir Cup Football Final of Qatar 2020? A call for action. Biol. Sport.

[5] Sparrow A.K. (2021). Protecting Olympic participants from Covid-19—the urgent need for a risk-management approach. N. Engl. J. Med..

[6] Sato S. (2022). The COVID-19 outbreak and public perceptions of sport events in Japan. Managing Sport and Leisure.

[7] Dergaa I. (2021). COVID-19 vaccination, herd immunity and the transition toward normalcy: challenges with the upcoming sports events. Annals of applied sport science.

[8] Shimizu K., Mossialos E., Shibuya K. (2022). What has the 2020 Tokyo Olympic and Paralympic Games taught global health on sporting mass gatherings under COVID-19 pandemic?. Anaesthesia Critical Care & Pain Medicine.

[9] WHO (2020). Status of environmental surveillance for SARS-CoV-2 virus. https://www.who.int/publications/i/item/WHO-2019-nCoV-sci-brief-environmentalSampling-2020-1.

[10] Radic A. (2021). Intention to take COVID-19 vaccine as a precondition for international travel: application of extended norm-activation model. Int. J. Environ. Res. Publ. Health.

[11] Diamond M.B. (2022). Wastewater surveillance of pathogens can inform public health responses. Nat. Med..

[12] Jiménez-Rodríguez M.G. (2022). Biosensors for the detection of disease outbreaks through wastewater-based epidemiology. TrAC, Trends Anal. Chem..

[13] Mao K. (2021). Biosensors for wastewater-based epidemiology for monitoring public health. Water Res..

[14] Li J. (2023). A global aircraft-based wastewater genomic surveillance network for early warning of future pandemics. Lancet Global Health.

[15] Haney J. (2022). Coinfection by influenza A virus and respiratory syncytial virus produces hybrid virus particles. Nature Microbiology.

[16] Pellegrinelli L. (2017). Surveillance of poliomyelitis in Northern Italy: results of acute flaccid paralysis surveillance and environmental surveillance, 2012–2015. Hum. Vaccines Immunother..

[17] Pellegrinelli L. (2022). Wastewater surveillance captured an increase in adenovirus circulation in milan (Italy) during the first quarter of 2022. Viruses.

[18] Administration U.F.D. (2020). Emergency use authorization. https://www.fda.gov/emergency-preparedness-and-response/mcm-legal-regulatory-and-policy-framework/emergency-use-authorization.

[19] Kitajima M. (2022). COVID-19 wastewater surveillance implemented in the Tokyo 2020 Olympic and Paralympic Village. J. Trav. Med..

[20] Saththasivam J. (2021). COVID-19 (SARS-CoV-2) outbreak monitoring using wastewater-based epidemiology in Qatar. Sci. Total Environ..

[21] El-Malah S.S. (2022). Application of human RNase P normalization for the realistic estimation of SARS-CoV-2 viral load in wastewater: a perspective from Qatar wastewater surveillance. Environmental Technology & Innovation.

[22] La Rosa G. (2020). First detection of SARS-CoV-2 in untreated wastewaters in Italy. Sci. Total Environ..

[23] Bibby K., Peccia J. (2013). Identification of viral pathogen diversity in sewage sludge by metagenome analysis. Environ. Sci. Technol..

[24] Monpoeho S. (2001). Best viral elution method available for quantification of enteroviruses in sludge by both cell culture and reverse transcription-PCR. Appl. Environ. Microbiol..

[25] Control C.f.D. (2020).

[26] Hasan M.R. (2019). A novel real-time PCR assay panel for detection of common respiratory pathogens in a convenient, strip-tube array format. J. Virol Methods.

[27] Sun H. (2021). Validation of a redesigned pan-poliovirus assay and real-time PCR platforms for the global poliovirus laboratory network. PLoS One.

[28] Zheng Q.-D. (2017). Estimating nicotine consumption in eight cities using sewage epidemiology based on ammonia nitrogen equivalent population. Sci. Total Environ..

[29] Been F. (2014). Population normalization with ammonium in wastewater-based epidemiology: application to illicit drug monitoring. Environ. Sci. Technol..

[30] Authority, Q.S. *Populatioon*. 2022 04042024]; Available from: https://www.psa.gov.qa/en/statistics1/StatisticsSite/Pages/Population.aspx.

[31] Altarawneh H.N. (2022). Effects of previous infection and vaccination on symptomatic omicron infections. N. Engl. J. Med..

[32] Chemaitelly H. (2023). Bivalent mRNA-1273.214 vaccine effectiveness against SARS-CoV-2 omicron XBB* infections. J. Trav. Med..

[33] Chemaitelly H. (2021). Waning of BNT162b2 vaccine protection against SARS-CoV-2 infection in Qatar. N. Engl. J. Med..

[34] Dergaa I. (2023). Large-scale sporting events during the COVID-19 pandemic: insights from the FIFA World Cup 2022 in Qatar with an analysis of patterns of COVID-19 metrics. Biol. Sport.

[35] WHO. Monthly operational update on health emergencies. December-2022 04042024]; Available from: https://www.who.int/publications/m/item/monthly-operational-update-on-health-emergencies-december-2022.

[36] Chemaitelly H. (2023). Turning point in COVID-19 severity and fatality during the pandemic: a national cohort study in Qatar. BMJ Public Health.

