# Supplementary material for: Leveraging wastewater surveillance for managing the spread of SARS-CoV-2 and concerned pathogens during FIFA World Cup Qatar 2022
Source: Heliyon. 2024 Apr 26;10(9):e30267. doi: 10.1016/j.heliyon.2024.e30267 (PMC11070812; doi:10.1016/j.heliyon.2024.e30267)
Supplement: Multimedia component 1 [file mmc1.docx]

**Supplementary Information**

**Leveraging Wastewater Surveillance for Managing the Spread of SARS-CoV-2 and Concerned Pathogens during FIFA World Cup Qatar 2022**

Shimaa S. El-Malah ^1^, Jayaprakash Saththasivam ^1^, Arun K K ^1^, Khadeeja Abdul Jabbar ^1^, Tricia A Gomez ^1^, Sara M. Wahib ^1^, Jenny Lawler ^1^ Patrick Tang ^2^, Faheem Mirza ^2^, Hamad Al-Hail ^2^, Khalid Ouararhni ^3^, Thasni K. Abdul Azis ^3^, Laith Jamal Abu Raddad ^4^, Hiam S. Chemaitelly^4^, Hussein A Abu Halaweh ^5^, Sara Khalife ^6^, Roberto Bertollini ^6^, Khaled A. Mahmoud ^1^*

^1^Qatar Environment and Energy Research Institute (QEERI), Hamad Bin Khalifa University, Qatar   Foundation, P. O. Box 34110, Doha, Qatar.

^2^Department of Pathology, Sidra Medicine, Doha, Qatar.

^3^Genomics Core, Hamad Bin Khalifa University, Qatar Foundation, Doha, Qatar.

^4^Infectious Disease Epidemiology Group, Weill Cornell Medicine-Qatar, Cornell University, Doha, Qatar.

^5^Drainage Network Operation & Maintenance Department; Public Works Authority, Doha, Qatar

^6^Ministry of Public Health, Doha, Qatar

*^*^Correspondence to: KA Mahmoud* [*kmahmoud@hbku.edu.qa*](mailto:kmahmoud@hbku.edu.qa)

Table S1. Sample distribution and number during this study

| **Duration** | **Frequent of Sampling** | **No. of Samples** |
| --- | --- | --- |
| **Pre-event**  **(23-30, Oct. 2022)** | Once per week | 10 |
| **During-event**  **(6 Nov. – 4 Dec. 2022)** | Twice per week | 40 |
| **Post-event**  **(13 Dec. 2022 - 29 Jan. 2023)** | Once per week only the first week twice | 55 |

Table S2. [Oligonucleotide](https://www.sciencedirect.com/topics/earth-and-planetary-sciences/oligonucleotide) sequences of primers and probes were used in this study ^[1-3]^.

| Assay | Target gene | Primer/probe | Sequence (5′–3′) |
| --- | --- | --- | --- |
| CDC N1 | Nucleocapsid (N) | 2019-nCoV_N1-F | GACCCCAAAATCAGCGAAAT |
|  |  | 2019-nCoV_N1-R | TCTGGTTACTGCCAGTTGAATCTG |
|  |  | 2019-nCoV_N1-P | FAM-ACCCCGCATTACGTTTGGTGGACC[^a^](https://www.sciencedirect.com/science/article/pii/S0048969721006768#tf0005) |
| CDC N2 | Nucleocapsid (N) | 2019-nCoV_N2-F | TTACAAACATTGGCCGCAAA |
|  |  | 2019-nCoV_N2-R | GCGCGACATTCCGAAGAA |
|  |  | 2019-nCoV_N2-P | FAM-ACAATTTGCCCCCAGCGCTTCAG[^a^](https://www.sciencedirect.com/science/article/pii/S0048969721006768#tf0005) |
| CDC RP | Human RNase P | RNAse P-F | AGATTTGGACCTGCGAGCG |
|  |  | RNAse P-R | GAGCGGCTGTCTCCACAAGT |
|  |  | RNAse P-P | FAM–TTCTGACCTGAAGGCTCTGCGCG[^a^](https://www.sciencedirect.com/science/article/pii/S0048969721006768#tf0005) |
| Human enterovirus | | HEV-F1 | CYG CRG CGG AAC CGA CTA C |
|  |  | HEV-F2 | CYG TGG CGG AAC CGA CTA C |
|  |  | HEV-R1 | GGA TGG CCA ATC CAA TAG CTA TAT |
|  |  | HEV-R2 | GGC CAA TCC AAH TCG CTT TAT |
|  |  | HEV-Probe | FAM-TTG GGT GTC CGT GTT T-MGB |
| Pan-poliovirus | | PanPV-F | TTG GAG TTC TTC ACI TAI TCI MGI TTY GAY ATG |
|  |  | PanPV-R | GGAGCTCCGGGTGGGAYRTACATIATYTGRTAIAC |
|  |  | PanPV-Probe | FAM-TGR TTN ARI GCR TGI CCR TTR TT-BHQ |

^a^FAM, 6-carboxyfluorescein;

Cross-correlation analysis (Table S3 and Fig S1) identified a significant positive correlation (r = 0.53234) at lag 0, indicative of a moderate in-phase relationship between clinical data and viral load. This signifies that changes in clinical data could coincide with changes in viral load. The Pearson correlation coefficient (r) was calculated to be 0.5324 (95% CI = 0.1540 to 0.7746), indicating a moderate positive correlation between the number of COVID-19 cases in clinical samples and the concentration of viral RNA detected in wastewater samples. The coefficient of determination (R squared) was 0.2835, suggesting that approximately 28.35% of the variability in clinical cases can be explained by changes in the viral load of wastewater. The two-tailed p-value was found to be 0.0089, which indicates that the observed correlation is statistically significant at the 0.05 level. These results highlight the potential of wastewater-based epidemiology as a complementary approach to clinical testing for monitoring COVID-19 prevalence in communities.

Table S3: Summary of Cross-correlation analyses and P value comparing clinical data and viral load time series.

| Correlation | Clinical Vs. Viral load |
| --- | --- |
| Pearson r |  |
| r | 0.5324 |
| 95% confidence interval | 0.1540 to 0.7746 |
| R squared | 0.2835 |
| P value |  |
| P (two-tailed) | 0.0089 |
| P value summary | ** |
| Significant (alpha = 0.05) | Yes |

Fig S1: Cross-correlation function for clinical cases and viral load

**References**

1. Control, C.f.D. and Prevention, *CDC 2019-novel coronavirus (2019-nCoV) real-time RT-PCR diagnostic panel.* 2020.

2. Hasan, M.R., et al., *A novel real-time PCR assay panel for detection of common respiratory pathogens in a convenient, strip-tube array format.* Journal of virological methods, 2019. **265**: p. 42-48.

3. Sun, H., et al., *Validation of a redesigned pan-poliovirus assay and real-time PCR platforms for the global poliovirus laboratory network.* PLoS One, 2021. **16**(8): p. e0255795.
